# IRBEVF-Q: Optimization of Image–Radar Fusion Algorithm Based on Bird’s Eye View Features

**DOI:** 10.3390/s24144602

**Published:** 2024-07-16

**Authors:** Ganlin Cai, Feng Chen, Ente Guo

**Affiliations:** 1 School of Computer and Big Data, Minjiang University, Fuzhou 350108, China; 2 College of Physics and Information Engineering, Fuzhou University, Fuzhou 350108, China

**Keywords:** 3D object detection, multimodal fusion, attention mechanism, query optimization, transformer

## Abstract

In autonomous driving, the fusion of multiple sensors is considered essential to improve the accuracy and safety of 3D object detection. Currently, a fusion scheme combining low-cost cameras with highly robust radars can counteract the performance degradation caused by harsh environments. In this paper, we propose the IRBEVF-Q model, which mainly consists of BEV (Bird’s Eye View) fusion coding module and an object decoder module.The BEV fusion coding module solves the problem of unified representation of different modal information by fusing the image and radar features through 3D spatial reference points as a medium. The query in the object decoder, as a core component, plays an important role in detection. In this paper, Heat Map-Guided Query Initialization (HGQI) and Dynamic Position Encoding (DPE) are proposed in query construction to increase the a priori information of the query. The Auxiliary Noise Query (ANQ) then helps to stabilize the matching. The experimental results demonstrate that the proposed fusion model IRBEVF-Q achieves an NDS of 0.575 and a mAP of 0.476 on the nuScenes test set. Compared to recent state-of-the-art methods, our model shows significant advantages, thus indicating that our approach contributes to improving detection accuracy.

## 1. Introduction

As autonomous driving vehicles continue to evolve, the integration of various sensors that are equipped to perceive the surrounding environment has become a vital aspect of the system’s operation [1]. However, achieving accurate and robust detection of 3D objects remains a challenge [2]. To address this, sensor fusion solutions [3,4,5,6,7] have been developed that aim to combine the strengths of different sensors, particularly cameras and LiDARs. While cameras are well suited for capturing rich texture and semantic information, LiDAR is effective at capturing spatial structural information [8]. Unfortunately, both sensors are prone to limitations in harsh weather conditions, such as heavy rain and snow. Though this combination of sensors can achieve better detection results, it is not sufficient for practical applications of autonomous vehicles. To overcome these limitations, millimeter-wave radar is a more robust sensor that can be used in all weather conditions [9]. Additionally, radar can provide critical velocity information through the Doppler effect, which is essential for collision avoidance in autonomous driving environments. Furthermore, radar sensors are relatively lower in cost and can complement the strengths of cameras and LiDARs. Despite these advantages, there has been limited research [10,11] focused on the fusion of radar data with other sensors. One significant challenge is the lack of datasets containing radar data for autonomous driving applications, thus making it difficult to conduct research in this area. Furthermore, applying existing LiDAR-based algorithms [12,13,14,15,16] to radar point clouds has proven to be highly challenging [17] due to the sparsity of radar point clouds, which makes extracting the geometric information of objects challenging.

Currently, the existing work [17,18] on fusing radar and cameras for 3D object detection employs a matching-based approach to accomplish the fusion. First, the view frustum generated by the camera is used to filter associated radar points. Then, the radar points are projected onto the camera image to generate radar features. These features are then concatenated with the image features to create associated radar–image features that are used for object detection. Another type of method uses the DETR paradigm [19,20,21,22,23,24], thus treating queries as the targets to be predicted, and it employs an attention mechanism and multilevel decoders for learning. Regarding these methods, we identify the following issues in radar and image fusion for 3D object detection: (a) the heterogeneity of image and radar features makes direct alignment impossible; and (b) the fusion methods or structures are overly simplistic, thus preventing full and deep integration. A major reason for these problems [25] is that images lack depth information, thus making it difficult to accurately correlate radar data with images. Since image data are in perspective space and radar data are in a 3D bird’s eye view (BEV) space, projecting the image into the radar feature space is ill posed. On the other hand, projecting radar points into the image feature space makes it extremely challenging to perform 3D perception tasks on a 2D image plane. To address this issue, new approaches are needed that can effectively fuse multimodal data while preserving the essential characteristics of each sensor. This requires developing new techniques that can accurately associate radar data with images while preserving the rich spatial information provided by radar.

To this end, we present a novel fusion scheme for 3D object detection using radar and camera data, which is termed the BEV fusion encoder. The BEV furnishes traffic scenes with relative localization and scale information and can be precisely mapped to the physical world, thus facilitating 3D object detection [26]. Furthermore, The BEV representation serves as a physical medium, thus offering an interpretable fusion approach for data from various sensors and timestamps and giving this scheme a significant edge over existing approaches [27]. The BEV fusion encoder that we propose not only elevates image features from 2D (two-dimensional) space to 3D (three-dimensional) space implicitly but also incorporates BEV-formatted radar data. Additionally, the BEV fusion encoding scheme decouples the network’s dependence on the correlation between images and radar while accomplishing the fusion of images and radar in an adaptive manner.

In the object decoder, a set of queries is used to learn information from the BEV features. The queries usually contain content queries and location embeddings. First, for the problem of random initialization of query content, this paper proposes heat map-guided query initialization HGQI, i.e., heat maps are generated based on the image features, and the peaks of the heat maps are used to initialize the object queries. Secondly, this paper proposes a dynamic position encoding module DPE for the problem of fixed query position encoding, which takes the output of each decoding layer as the position encoding input of the next layer, thus providing more effective a priori information for the position encoding of the query. In addition, the query also participates in the process of positive and negative sample matching, but the model convergence is difficult due to the instability of bipartite graph matching. Therefore, this paper also employs an auxiliary noise query module, ANQ, to help stabilize the matching problem during training by adding noise queries with known ground truth values to make the object query focus on the regression of the location information.

The key contributions of our works are as follows:

- We compared various radar and image fusion schemes for 3D object detection, summarized the current research shortcomings, and identified the underlying reasons.
- The BEV fusion encoder is proposed. We simulated 3D spatial reference points and learned the intrinsic connection between radar and images through a multilayer structure to generate BEV features with good 3D spatial perception.
- We optimized the decoder structure. We generated heat maps for initial content query through a priori information, and we additionally utilized dynamic correction of the reference points to improve position coding. Then auxiliary noise was used in the training phase to help stabilize the convergence.

## 2. Materials and Methods

### 2.1. Architecture

**Figure 1:**
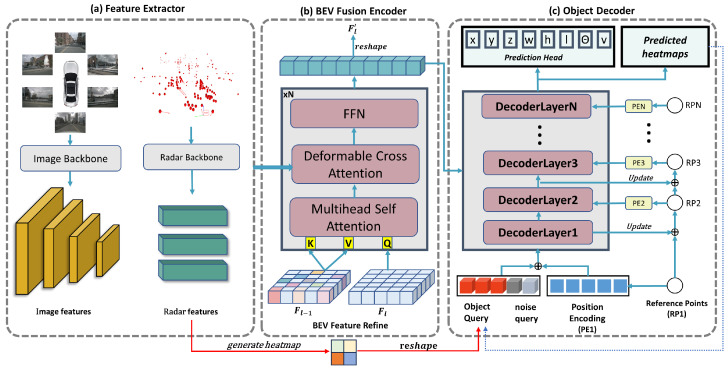
The overall architecture of our implementation of 3D object detection consists of three main components: (**a**) Feature extraction module. (**b**) BEV fusion encoder module. The input radar and image features are encoded mainly through local image sampling attention and maximum adjacent radar sampling attention to generate BEV features. (**c**) Object decoder module. Content encoding is initialized using heat maps, and position encoding is generated using dynamic reference points. In addition, noisy queries are added to the query to help stabilize the matching process.

As shown in Figure 1, the proposed model in this paper mainly consists of a feature extraction module, a BEV fusion encoder, and an object decoder. In the training phase, we extract features from the multiview images and radar data separately using the feature extraction module. Specifically, given N input images $I\in R^{N\times 3\times W\times H}$ at one time, the multiscale image features $F_{\mathrm{cam}}\in R^{N\times L\times c\times w\times h}$ are obtained by ResNet101 [28] with an *L* layer FPN [29]. For the raw radar data, the coordinates, velocity, relative velocity, and radar cross-section (RCS) are fed into a multilayer perceptron to obtain the $D_{1}$ dimensional feature Radar Feature $F_{\mathrm{rad}}\in R^{M_{1}\times D_{1}}$. The image feature and radar feature are fed into the BEV fusion encoder, which is updated by stacking multiple coding layers to obtain the BEV feature. Finally, the decoder uses multiple query embeddings for the BEV feature and uses two feedforward neural (FFN) networks to decode the information from the query to obtain the category and bounding box predictions.

### 2.2. BEV Fusion Encoder

For the BEV fusion encoder, we follow the encoder structure of Transformer [30], which consists of *l* encoding layers. We predefine a set of learnable parameters $F_{\mathrm{BEV}}\in R^{M_{2}\times D_{2}}$ as the initialization of BEV features, where $M_{2}$ represents the size of flattened features, which should be similar to the product of the width and height of the image, and $D_{2}$ represents the dimension of each BEV feature. Then, the BEV features of the current layer are subjected to multihead self-attention (MSA) with the BEV features output from the previous layer. The resulting BEV features are then processed by multihead crossattention(MCA) and an FFN network to obtain the output of this encoding layer.

**Figure 2:**
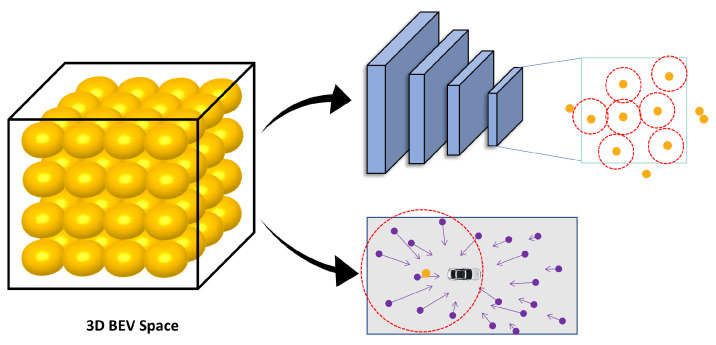
The details of BEV Fusion Encoder. Discretizing three-dimensional space into uniformly distributed points, project these points onto a multiscale feature map, and sample the points that fall into the feature map. In addition, the distance of these points from the radar point is calculated, and each point is sampled for the closest radar feature.

As shown in Figure 2. The BEV fusion coding module is mainly used to generate BEV features with 3D spatial awareness by aggregating two different modal features, namely image features and radar features. It includes local image sampling module, maximum proximity radar sampling module, and BEV feature generation module.

**Figure 3:**
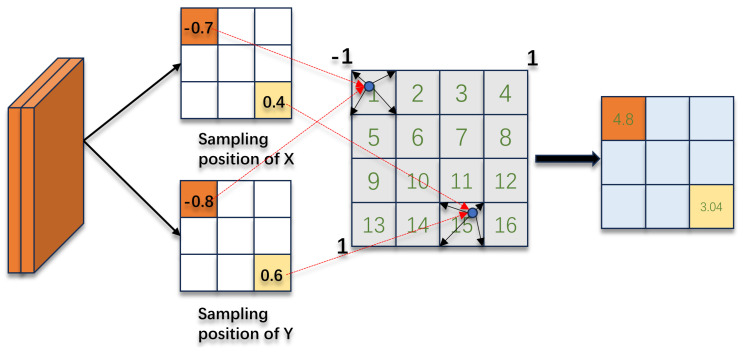
Local image feature sampling. Project the 3D reference point onto the graph to obtain the sampling position in the x and y directions. Obtain the characteristic value of the sampling point by obtaining the values around the sampling point.

As shown in Figure 3, in the multihead crossattention, we encode the image and radar features separately using deformable attention [31] and minimum distance sampling. First, the BEV space is discretized into a set of points $P\in R^{N\times 3}$ [32]; these points are projected using camera transformation matrices to obtain their relative positions $P_{cam}$ in multicamera multiscale image features. Then, deformable attention is applied to sample the relevant image features, which can be represented as follows:

(1) $$F_{bev\_cam}=\frac{1}{N_{p}}\sum_{i=0}^{N}\sum_{j=0}^{L}\;\mathrm{DeformAttention}\;\left(F_{i,j},P_{\mathrm{cam}\;}\right)$$

where *N* represents the number of cameras, *L* represents the number of feature maps at different scales, $N_{p}$ denotes the number of points projected onto the images, and $F_{i,j}$ represents the jth feature map of the ith camera.

**Figure 4:**
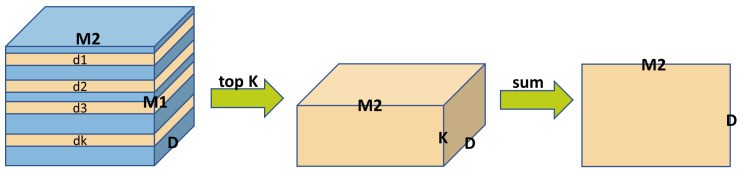
Maximum proximity radar sampling. The distance between the radar point and the 3D reference point is calculated to obtain a 3D tensor. The features of the K radar points closest to each 3D reference point are taken as sampling features.

For the BEV encoding of radar features, we normalize the coordinates of radar points in BEV space and calculate the Euclidean distance between them and the point set in the BEV space. For each point in the BEV space, we select the K radar points with the smallest distance and use the sampled radar information to generate BEV features encoded by radar. As shown in Figure 4, for $M_{1}$ spatial points and $M_{2}$ radar points, the radar features sampled by each spatial point can be obtained based on the indices of the calculated distances. The D−dimensional feature corresponding to each of the $M_{1}$ spatial points can be obtained by summing over the K dimensions. This sampling process can be represented as $F_{sample}$:

(2) $$F_{bev\_rad}=F_{sample}\left(F_{rad}\right)$$

The image features sampled in BEV space are combined with radar features to obtain BEV features through a neural network:

(3) $$F_{BEV}=\Phi \left(F_{bev\_cam}⊕F_{bev\_rad}\right)$$

where Φ(·) stands for multilayer perceptron, and ⊕ represents two tensors concated together in the last dimension. The output BEV features are first subjected to a self-attention operation with the BEV features of the next layer. The purpose is to extract the spatial positional information of each feature from the previous layer’s BEV output, thus providing prior knowledge for subsequent sampling tasks. For the first BEV feature encoding layer, let the current BEV features perform the self-attention operation among themselves.

### 2.3. Decoder and Query Optimization

The decoder was designed using the DETR [33] paradigm by setting a set of spatial object queries $Q^{l}={\left\{q_{i}\right\}}_{i=1}^{N_{q}}\in R^{N_{q}\times D_{q}}$, where $N_{q}$ denotes the number of queries, and $D_{q}$ denotes the dimension of each query. $Q^{l}$ contains both content queries and location queries.In this paper, we improved the decoder structure by optimizing the query.

#### 2.3.1. Heat Map Guide Query Initial

In object detection tasks, heat maps can help identify the locations and regions of objects, thus indicating which regions the model believes contain specific objects. Therefore, we used heat maps to provide prior information and initialize query content embedding. We used radar to generate heat maps during the first training. For generating heat maps using radar BEV features, the procedure is as follows: Give the radar feature map $R_{BEV}\in R^{C_{rb}\times H_{rb}\times W_{rb}}$, where $C_{rb}$, $H_{rb}$, and $W_{rb}$ represent the channel number, height, and width of the radar feature map, respectively. Initially, a fully connected layer is utilized to transform the channel number of the radar feature map into the number of classes $C_{l}$ that the network needs to predict. Subsequently, each value in the radar feature map is normalized to the range of 0−1. Next, we have a tensor $L_{MAX}\in R^{C_{rb}\times H_{rb}\times W_{rb}}$, where $C_{rb}$ of the same size as the radar feature map is established to acquire local maxima, thus following the rule that each value must be greater than the surrounding 8 values. The values of $R_{BEV}$ are then compared to the values of $L_{MAX}$ one by one, and if they are the same, the maximum value is retained, thus selecting the top−K maximum values in descending order. Finally, the two−dimensional feature map is folded into a one−dimensional vector, thus obtaining the index of each maximum value on the one-dimensional vector. Using these position indices on the folded original radar BEV feature map, the values of the original radar BEV feature map are obtained. At this point, a heat map $H\in R^{C_{rb}\times K}$ is given, where $C_{rb}$ is generated from the radar BEV feature map, and through transposition, the query $Q\in R^{K\times M}$ initialized by the heat map can be obtained, where M represents the dimension of each query, which is equivalent to the size of $C_{rb}$. An additional output head is added at the output of each decoding layer for predicting the heat map $H_{pre}\in R^{K\times H_{rb}\times W_{rb}}$.

For the generation of the ground truth of the heat map, firstly, the number of objects with a ground truth is obtained from the ground truth labels, and the width h and height w of the object frame are obtained from the ground truth label of each object, which are used to compute the radius r. It is still mainly dependent on the overlap between the real frame’s and the predicted frames, and the value of the radius r is taken according to the different critical situations.

According to the form of the solution of the binary equation, three radii $r_{1}$, $r_{2}$, and $r_{3}$ can be obtained by IOU:

(4) $$IOU_{1}=\frac{h*w}{\left(h+2r_{1}\right)*\left(w+2r_{1}\right)}$$

(5) $$IOU_{2}=\frac{\left(h-2r_{2}\right)*\left(w-2r_{2}\right)}{h*w}$$

(6) $$IOU_{3}=\frac{\left(h-r_{3}\right)*\left(w-r_{3}\right)}{2*h*w-\left(h-r_{3}\right)*\left(w-r_{3}\right)}$$

Taking the minimum of $r_{1}$, $r_{2}$, and $r_{3}$ is the required radius r.

Next, use the obtained for radius r to establish the rectangular grid coordinates of rectangles −r−r. For each coordinate, apply the Gaussian function $G=e^{-\frac{x^{2}+y^{2}}{2\sigma^{2}}}$ to obtain the Gaussian distribution of the rectangles in order for the values spreading out in all directions from the center of the object to decay according to the distance for penalizing long-range prediction. Then, obtain the center of the object (x, y) from the real value label, obtain the size of the top, bottom, left, and right that each center can cover according to the width, height, and radius of the heat map, and crop the Gaussian distributed rectangle to the center of each object, and this completes the generation of the real value of the heat map.

#### 2.3.2. Dynamic Position Encoding

Positional encoding is a crucial concept in image tasks, particularly within the Transformer architecture. Due to its self-attentive mechanism, the Transformer inherently lacks order perception of the elements in a sequence. Consequently, without positional encoding, the Transformer cannot distinguish the relative positions of the elements. Positional encoding enhances the model’s expressive power by providing additional information, thus enabling better sequence understanding and generation. Moreover, positional encoding is learnable, thus allowing the model to adapt during training to better suit specific tasks.This section introduces improvements to the traditional positional encoding in the Transformer architecture for the task of 3D object detection. Unlike traditional positional encoding, which uses random parameters to learn positional information from the query, the dynamic positional encoding proposed here leverages positional information connected with the predicted values (x, y, z) of the outputs from each layer. This approach provides more accurate positional information related to the objects.

**Figure 5:**
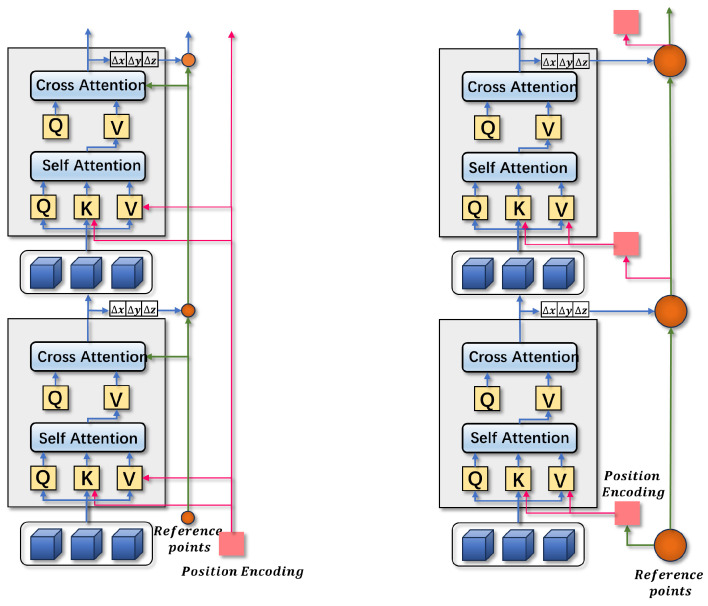
Original position encoding and dynamic position encoding. Traditional position coding uses randomly generated fixed position coding to generate reference points. Dynamic position coding first generates reference points then uses the reference points to generate position coding. At the same time, each layer outputs position information to modify the reference points, so the position coding information also changes dynamically.

A comparison with the structure of conventional position coding is illustrated in Figure 5. For the reference point $Ref\in R^{K\times 3}$, which is used in detection models as a crucial medium for the interaction of different modalities, the previous algorithm employed randomly initialized position encoding information $Pos\in R^{K\times M}$. In contrast, the proposed dynamic position encoding algorithm first initializes the value of the reference point, then utilizes this reference point to generate position encoding information, and subsequently uses the output of each layer to continuously optimize the position of the reference point. This process ensures that the position information of the reference point more closely aligns with the position of the real object. By using the position encoding generated from the reference point, a positional prior is provided for the subsequent decoding of the query, thus allowing the query to search for the position of the real object more accurately. The use of reference points to generate position encoding for query decoding provides a positional prior that enables the query to begin its search from a position relatively close to the actual object position. This approach results in faster convergence and improved performance compared to the traditional position encoding method, as it reduces the solution space and thereby lessens the breadth and complexity of the neural network’s parameter learning.

Then, for the obtained reference point above, it first passes through a sinusoidal encoding module:

(7) Enc(Ref)=sinRef/10,0002iM/2cosRef/10,0002i+1M/2

where M is the dimension representing the position encoding, the new reference point $Ref_{1}\in R^{K\times 3M/2}$ is obtained by sinusoidal encoding, and then the reference point $Ref_{2}\in R^{K\times M}$ is obtained by a fully connected layer F with the parameter (3M/2,M). This algorithm also sets a scale function Scale for the MLP multilayer perceptron with parameters (M, 2M, M) for generating ${Pos}_{scale}$ for sensing object scale information for position encoding, where the scale function ${Pos}_{scale}$ is 1 for the first of the L decoding layers and ${Pos}_{scale}$ for the other layers. The output of a layer is generated by the scale function Scale, and the final position encoding Pos is the product of $Ref_{2}$ and ${Pos}_{scale}$.

#### 2.3.3. Auxiliary Noise Query

The decoder addresses target detection as an ensemble prediction problem, thus aiming to achieve an optimal matching between each predicted frame and the true frame that minimizes the overall cost. The instability of the cost matrix results in unstable matching between the query and ground truth, which frequently disrupts the learning process of the query. To alleviate this problem, this paper introduces an auxiliary noise query module using the true value information to stabilize the matching process and optimize the learning conditions of the query.

In this paper, the query is divided into two parts. The first part, the matching component, follows the same processing method as the query in the previous model, thus using the Hungarian algorithm for matching and learning to approximate the true value labeled pairs and using the matched decoder outputs. The second part is the denoising component. The input for this component is the noisy ground truth, and the output aims to reconstruct the real 3D frame object. Since the added noise is relatively small, it is easier for the model to predict the corresponding GT based on these noisy inputs, thereby reducing the learning difficulty. Additionally, because the learning goal is clear, the input derived from a specific noised GT will be responsible for predicting its corresponding true value, thus avoiding the dichotomous phenomenon introduced by Hungarian matching.

**Figure 6:**
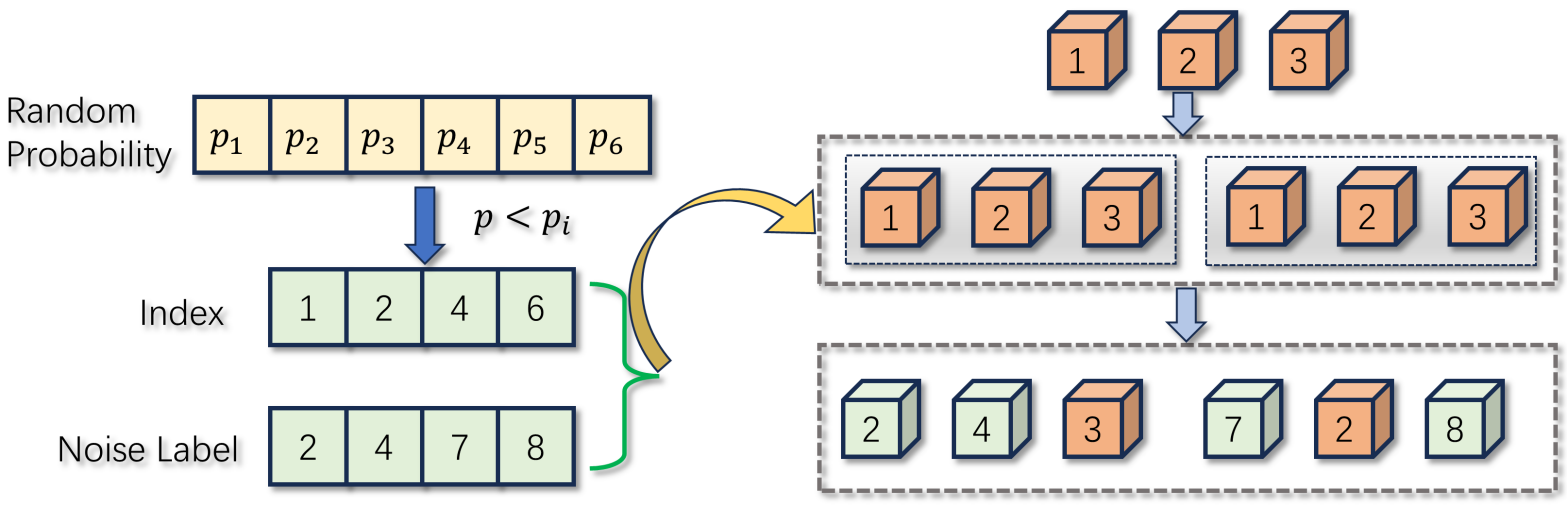
Original position encoding and dynamic position encoding. Generate noise labels and indexes through probability, and disrupt the labels of ground truth through indexes.

The construction of the denoising query consists of two main components: labeling noise and 3D box noise. Additionally, this algorithm applies various noise addition scenarios to the ground truth (GT), as illustrated in Figure 6. Specifically, assuming a batch of data contains d real GT values repeated n times to construct n groups of different denoising queries, the total number of denoising queries is n×d, which is denoted by D. For label noise addition, the algorithm first generates D probability values conforming to a normal distribution. It then uses a preset probability p to screen k indexes of D probability values that are smaller than p. Next, k positive integers representing random labels within the range of the number of categories are generated. These k random labels are then adjusted according to the indexes, thus modifying the values at the real label locations after n repetitions to the labels with added noise. Finally, a fully connected layer encodes this noise query to obtain the noise query $Q_{Noise}\in R^{D\times M}$.

Finally, the original content query and the noise query are spliced in the number dimension to obtain the hybrid content query $Q_{hybridcontent}\in R^{\left(K+D\right)\times M}$.

For 3D box noise addition containing six parameters $\left(x,y,z,w,h,l\right)$, the main operations of this algorithm are centroid displacement and scale scaling, while to ensure small perturbation, the 3D frame parameters are first normalized to 0−1. For centroid displacement, 1 perturbation parameter is first sampled from a uniform distribution $lambda_{1}\in \left(0,1\right)$, and then the offset corresponding to the center point $\left(x,y,z\right)$ is caluclated as follows:

(8) $$\left|\Delta x\right|=\lambda_{1}x,\;|\Delta y|=\lambda_{1}y,\;\left|\Delta z\right|=\lambda_{1}z$$

and this constrains

(9) $$|\Delta x|<\frac{\lambda_{1}w}{2},|\Delta y|<\frac{\lambda_{1}h}{2},\left|\Delta z\right|<\frac{\lambda_{1}l}{2}$$

Similarly, 1 perturbation parameter $\lambda_{2}\in \left(0,1\right)$ is sampled from the uniform distribution, and then also the offset corresponding to $\left(w,h,l\right)$ is computed separately:

(10) $$\left|\Delta x\right|=\lambda_{2}w,\;|\Delta y|=\lambda_{2}h,\;\left|\Delta z\right|=\lambda_{2}l$$

The length, width, and height of the final 3D box is scaled to the interval [0, 2]. According to the dynamic position encoding algorithm above, it is known that the noise-added 3D box represents the reference point, which needs to be used to generate the position encoding, while the subsequent query of the decoder is the superposition of the content query and the position encoding.

After completing the design of the noise query, the current query has been changed to the hybrid query $Q_{hybrid}\in R^{\left(K+D\right)\times M}$; this query subsequently acts in the same way as the usage DETR model, but in the decoder, the hybrid query $Q_{hybrid}$ needs to go through the process of going through a self-attention module, which performs a global interaction. Thus, the query used for matching fetches the content of the noise query, which leads to information leakage, since the noise query is noised from the true-value GT information. Therefore, this section designs a mask module, which serves two purposes: firstly, it prevents the query used for matching from interacting with the noise query for information, and secondly, it prevents the noise query from interacting with the information between different groups. This ensures that the matching task and the denoising task are independent of each other and do not interfere with each other. Also, whether the denoising part can see the matching part does not affect the performance, because the queries in the matching part are learned queries and do not contain information about the GT objects.

This algorithm uses $Mask={\left[mij\right]}_{A\times A}$ matrix to represent the self-attention mask, where A=K+n×d, K is the number of queries used for matching, d is the number of true-valued GTs corresponding to this set of queries, and n represents the different denoising groups; if $m_{ij}=1$, it means that the ith query can interact with the jth query; if $m_{ij}=0$, it means that the ith query cannot interact with the jth query. As shown in Figure 4, the first K rows and K columns of the self-attention mask represent the matching part, and the subsequent K + d rows and K + d columns represent the part of a denoising group, of which there are n, and so on. The final noisy query prediction yields categories with 3D box sizes that still require the computation of the loss ${loss}_{AN}$:

(11) $$\begin{array}{cc} {\mathrm{loss}}_{AN} & =\lambda_{AN}*{\mathrm{loss}}_{AN_{CLS}}+\lambda_{AN}*{\mathrm{loss}}_{AN_{BOX}}\\ \; & =\lambda_{AN}*{\mathrm{focal}}_{loss}\left(P,G\right)+\lambda_{AN}*S_{L1}\left(P,G\right) \end{array}$$

where $\lambda_{AN}$ is used to control the weight of the auxiliary noise loss, *P* represents the predicted value, *G* represents the true value, and $S_{L1}$ represents the Smooth L1 loss, and since the auxiliary noise is noise added to the true value, the values of *P* and *G* are one-to-one, without the need to perform a positive–negative sample matching, which is theoretically equivalent to increasing the proportion of the positive samples, which is an intrinsic part of the validity of the approach This is also the intrinsic reason for the effectiveness of the method.

## 3. Results

### 3.1. Experimental Settings

Datasets: We carried out experiments on the nuScenes [34] dataset, which is the sole dataset that provides an abundance of multiview image and large-scale radar point cloud data, as shown in Table 1. We exclusively used keyframes in our experiments. The official evaluation metrics employed in our study encompass a wide range of performance indicators, including the mean Average Precision (mAP), mean Average Translation Error (mATE), mean Average Scale Error (mASE), mean Average Orientation Error (mAOE), mean Average Velocity Error (mAVE), mean Average Attribute Error (mAAE), as well as the nuScenes Detection Score (NDS). NDS integrates the above metrics by computing a weighted sum, with a weight of 5 assigned to the mAP, a weight of 1 assigned to each of the five error indicators, and the normalized total sum being calculated.

Model Details: In this experiment, the original size of the input image for the network is 1600 × 900. Through the feature extraction networks of Res101 and FPN, the 3−channel image was transformed into a feature map with channel sizes of 2048, 1024, 512, and 256. For radar data containing 14 dimensions of information, we used a multilayer perceptron to uniformly transform the radar features into 64 dimensions. Subsequently, the radar and image features were encoded to obtain transformed BEV features. These features have an initial size of 40,000, a feature dimension of 256, and a BEV feature resolution size of 0.512 m. A learnable position embedding was introduced for the BEV features, and the BEV fusion encoder consists of six encoding layers, thus refining the BEV features in each layer. The decoder has 900 object queries by default and contains six decoding layers. Finally, two network branches were used to predict the bounding box parameters and class labels for each object query. Each branch includes two fully connected layers, with a hidden dimension of 512 and an output dimension of 10.

Implementation Details: We took a multicamera 3D object detector and pretrained it as the pretraining weights for our camera-radar fusion network. We kept the weights of the camera feature extraction module frozen during the training of the camera-radar fusion network, while the other fusion modules were trained in an end-to-end fashion. The AdamW optimizer was used to train the model, with an initial learning rate of 2 × $10^{-4}$, and the learning rate was adjusted using the Cosine Annealing algorithm. During the first 500 steps, the learning rate was linearly increased from one-third of the initial learning rate to the initial learning rate and then decreased to 1 × $10^{-3}$ of the initial learning rate. The model was trained for a total of 24 epochs on four RTX 3090 GPUs, with a batch size of 1 for each GPU. For the AND module proposed in this paper, the hyperparameters set the number of denoising groups n to 5, the screening probability p to 0.2, and the auxiliary noise weights $\lambda_{AN}$ to 0.75.

### 3.2. Main Results

The baseline model for this experiment is referred to as IRBEVF, and the improved model proposed through this experiment will be referred to as IRBEVF-Q. In order to validate the effectiveness of the methods proposed in this section, this experiment compares the radar-image fusion model with state-of-the-art 3D object detection methods on both the nuScenes test set and the validation set, and only those with a paper or code on the nuScenes Detection Challenge Leaderboards have been used for comparison. This work serves as a comparison and used the data provided on the original paper.

As demonstrated in Table 2, employing the same ResNet101 backbone network on the nuScenes test set, our approach surpassed recent competitive camera-radar and multicamera methods. When compared to the benchmark model IRBEVF-Q, the proposed enhancement scheme in this paper yielded improvements of 4.0% and 4.6% in the primary metrics NDS and mAP, respectively. Additionally, enhancements were observed across all five error metrics, with a notable 6.5% reduction in the distance error, which is an area where the benchmark model underperformed. This improvement can be attributed to the theoretical benefits of dynamic location encoding, which enables the query to concentrate more on location information regression, thereby reducing prediction errors. In comparison to the highly effective camera-radar fusion method CRAFT, our method achieves a 5.2% enhancement in NDS and a 6.5% improvement in the mAP. Moreover, compared to the fusion method RCBEV, which also utilizes a BEV scheme, our method achieved a 7.1% improvement in the NDS and a 7.6% enhancement in the mAP. The significant performance gains in scale and velocity estimation errors, where velocity information is derived from radar data availability, underscore the effectiveness of the radar-camera fusion method proposed in this paper. Furthermore, the improvements in scale and position errors indicate that the query optimization and structural enhancements proposed in this section lead to a more focused iteration of query information, thus benefiting model prediction and convergence. These advancements will be further illustrated in the experiments discussed in the subsequent subsection.

The comparisons on the validation set are presented in Table 3. Compared to the benchmark model IRBEVF, the proposed enhanced model in this paper achieved improvements of 3.7% and 3.6% in the NDS and mAP, respectively. Additionally, enhancements were observed in the mATE, mASE, mAVE, and mAAE, with gains of 11.0%, 0.5%, 1.5%, and 4.9%, respectively, with the most significant improvements seen in the reduction of the position estimation errors. Compared to CRAFT, our model demonstrated gains of 5.1% and 4.7% in key metrics, with the error in distance estimation further reduced from 20.4% to 9.4%, while the error in speed estimation slightly increased from 15.6% to 17.1%. Furthermore, when compared to the simultaneous BEV solution RCBEV, the method proposed in this paper outperformed in terms of the NDS and mAP by 7.1% and 7.6%, respectively, and it reduced the speed estimation error by 15%. These outstanding results on both the validation and test sets underscore the effectiveness of the model enhancements proposed in this paper.

Table 4 compares the camera-only benchmark methods, namely Centernet, CRAFT-I, and IRBEVF, with the camera-radar fusion methods, including Centerfusion, CRAFT, and IRBEVF-Q, in terms of the improvement in the mAP for each category on the validation set. It is evident that the overall enhancement range of radar and image fusion-based Centerfusion over image-based Centernet was approximately 5%, and Centerfusion’s performance fell behind that of CRAFT with IRBEVF-Q comprehensively. CRAFT demonstrated a significant improvement in detecting large vehicles such as cars, trucks, and buses. However, its performance enhancement was limited for small objects such as pedestrians, bicycles, traffic cones, and obstacles, with even negative improvement observed for traffic cones. The method proposed in this section, IRBEVF-Q, not only achieved good detection results for large objects like cars, trucks, and buses, with a performance gap from CRAFT that is not excessively large, but it also exhibited superior performance and improvement in detecting small objects where CRAFT struggled. Experimental results demonstrate that the BEV representation-based method offers global representation, thus overcoming object occlusion issues arising from the lack of depth in pure vision and addressing challenges in small object detection in images. Utilizing BEV features as a fusion medium for image and radar demonstrates greater potential than traditional fusion methods. Furthermore, prediction based on the query obviates the need for postprocessing to propose redundant prediction frames, thus highlighting the advantages of the end-to-end model.

### 3.3. Ablation Study

In this section, experiments are conducted on the nuScenes validation set for validating the effectiveness of each module in the proposed improved algorithm. The experiments were conducted uniformly using 1600 × 900 images, the res101 backbone network, and pretrained loaded weights to train the model for 24 epochs.

Table 5 examines the influence of image, radar, and their fusion methods on 3D target detection. A comparison between scenarios (a) and (b) in the table reveals that the inclusion of radar information marginally enhanced the performance of 3D target detection. However, the improvement resulting from the mere addition of radar information was limited, thus emphasizing the necessity to identify a more effective fusion method to facilitate better learning by the neural network. Comparing schemes (b) and (c) demonstrates that the correlation between the radar and image data led to further performance enhancement through projection. Meanwhile, comparing schemes (d) and (b) underscores the effectiveness of the BEV fusion proposed in this paper for integrating image and radar information. It highlights that simple projection fails to fully exploit the inherent relationship between radar and image features.

Table 6 examines the impact of HGQI, DPE, and ANQ, as proposed in this paper, on the overall model. For standalone cases (b), (c), and (d), there was an improvement of 0.7%, 0.9%, and 1.3%, respectively, compared to the baseline model, thus indicating that each module can positively influence the baseline model. In the combined scenarios (e), (f), and (g), where two modules were paired, improvements of 2.0%, 2.8%, and 2.2% were observed, respectively, compared to the benchmark model. When comparing the combination of HGQI and ANQ (f) to the individual HGQI and ANQ modules, improvements of 2.1% and 1.5%, respectively, became evident. This suggests that overcoming query initialization randomness and ensuring query stability and matching positively affects the query-based 3D object detection scheme. For scenarios (e) and (g), both of which included the DPE module, its inclusion led to a stable improvement (1.3% performance enhancement for both) compared to using the HGQI and ANQ modules alone. This indicates that dynamic position coding can effectively alleviate the hindrance posed by the original fixed position coding in 3D object detection. Finally, with the simultaneous operation of all three modules, it was observed that once the randomness in the model leading to instability was mitigated, the model performance was proportionally enhanced. This reflects the fact that the improvements proposed in this paper can achieve synergistic effects, thus resulting in a performance boost greater than the sum of individual improvements.

As an important hyperparameter, the number of BEV features controls both the size and accuracy of the model. The experiment shown in Table 7 demonstrates that the model’s performance improved with the number of BEV features until it reached 40,000. Beyond that point, increasing the number of BEV features only increased the number of parameters without effectively improving performance.

Table 8 displays the variation in the performance and parameters of IRBEVF-Q across different numbers of decoding layers. The table reveals that the model performance improved as the number of decoding layers increased from one to six. However, the magnitude of improvement diminished, and the model performance declined when the number of decoding layers reached seven, thus suggesting that performance saturates at six layers. Furthermore, the performance of the IRBEVF-Q model matched that of the IRBEVF model when the number of decoding layers was three. This suggests that the proposed improvement module enables the model to achieve comparable performance with fewer decoding iterations.

**Figure 7:**
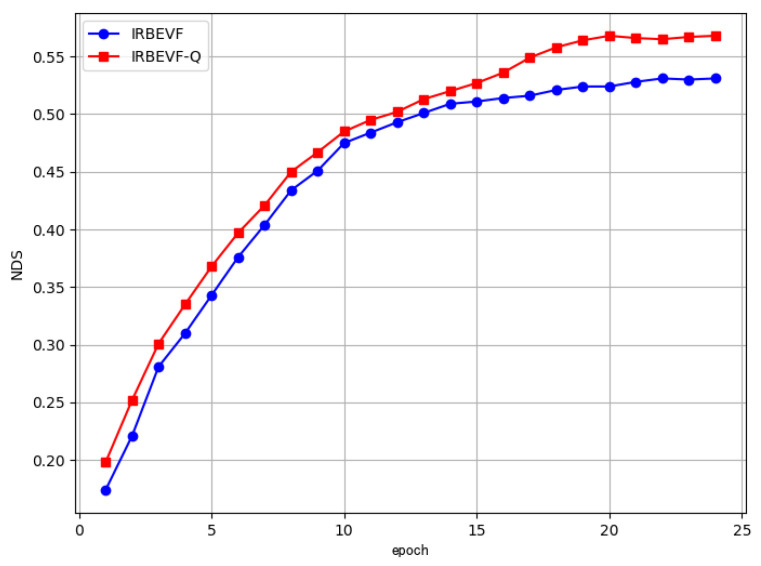
Comparison of the performance of different training epochs.

Figure 7 illustrates the NDS performance curves of the IRBEVF model and the IRBEVF-Q model. It is evident that the IRBEVF-Q model achieved peak performance by the 20th epoch, with subsequent fluctuations remaining near the peak performance. This indicates that the IRBEVF-Q model surpasses the IRBEVF model in terms of both performance and convergence speed. This improvement can be attributed to the enhanced model stability achieved through query optimization, which mitigates the impact of randomness on the model.

**Figure 8:**
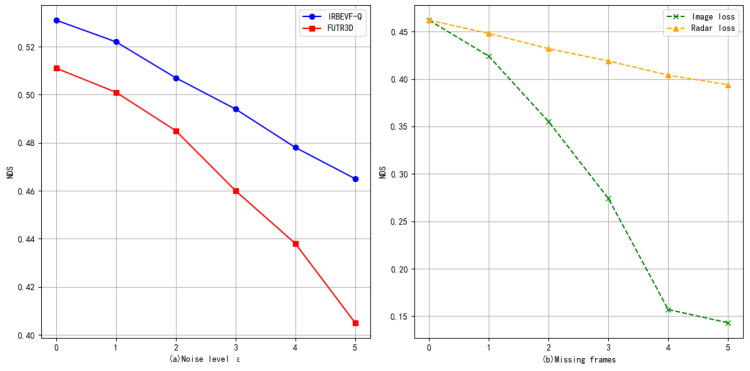
Results of ablation experiments with different noise levels (**a**) and sensor faults (**b**).

Noise and sensor failures are critical considerations in the operation of autonomous vehicles. We deliberately introduced noise and simulated experiments to address passive sensor failures. As seen in Figure 8a, noise was added to the projection matrix, with higher noise levels resulting in increased noise. Subsequently, as seen in Figure 8b, experiments involved the removal (or filling with zero matrices) of six image inputs and five radar inputs. As depicted in Figure 8a, when employing IRBEVF-Q and another outstanding 3D detection approach, FUTR3D, we observe that higher errors in the camera’s external reference led to more significant performance degradation. However, for the same level of noise error, the performance degradation of IRBEVF-Q compared to FUTR3D was less pronounced, thus indicating that IRBEVF-Q exhibits greater resilience to interference and enhancing the model’s robustness. From Figure 8b, we observe that both the loss of image frames and the loss of radar frames impacted the model’s performance. However, the impact of losing image frames is considerably larger than that of losing radar frames. This discrepancy arises from the high resolution and rich semantic information provided by images, which are crucial for model performance, whereas radar signals, with their lower resolution, serve primarily as supplementary information.

### 3.4. Visualization and Analysis

**Figure 9:**
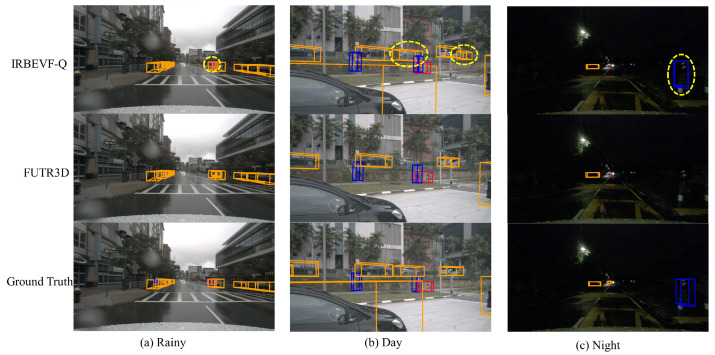
Comparison of 3D detection effect of different methods under different scene columns. The orange, blue, and red 3D boxes represent the predictions for cars, pedestrians, and bicycles, respectively. The yellow dashed ovals represent different places to watch out for.

**Figure 10:**
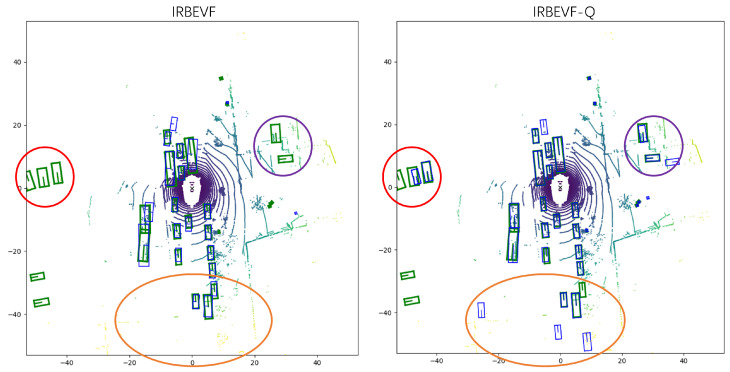
Comparison of BEV detection effect before and after model improvement. Red circles represent accuracy contrasts predicted at a distance, and orange circles represent contrasts predicted for potentially possible objects.

In Figure 9, we compare and analyze the 3D detection results of the open source radar–vision fusion detection work FUTR3D and the IRBEVF-Q model in this paper in the same scene. (a) In rainy days and nights when the camera image is not clear, our IRBEVF-Q can predict more and more effective information. First, the radar information brings more potentially useful features, and second, the radar image fusion method has a better effect. (b) In sunny weather, our IRBEVF-Q is more accurate in predicting occluded and truncated objects. The distance information brought by the radar brings a gain to the prediction after passing through our fusion method, which better overcomes the disadvantage of the image in predicting occluded objects. Figure 10 further contrasts the detection results of IRBEVF and IRBEVF-Q in the BEV view. Notably, IRBEVF-Q exhibited more accurate location predictions compared to IRBEVF, and it successfully anticipated a greater number of potential objects at a distance, thus facilitating road comprehension for autonomous vehicles.

**Figure 11:**
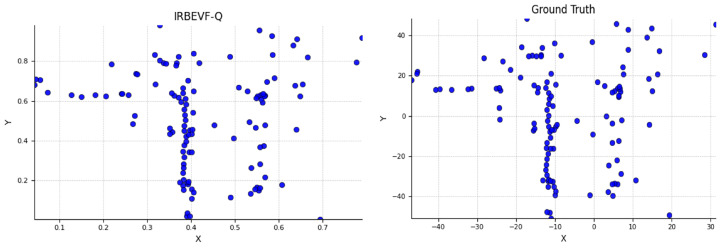
Schematic representation of the improved query location distribution.

Figure 11 illustrates the initial position distribution of the query in IRBEVF-Q during training, which is juxtaposed with the BEV coordinates within the true value box. It is evident that the relative position distribution of IRBEVF-Q within the range of 0 to 1 closely resembles the object distribution in the real coordinate system, thus spanning from −51.2 to 51.2. This alignment indicates that dynamic position coding enables the query to mitigate the adverse effects of random initialization during training.

## 4. Conclusions

This paper introduced a novel BEV fusion encoder, which leverages radar and image fusion to aggregate 3D spatial points and generate BEV features. Subsequently, we developed the 3D target detection model IRBEVF. Recognizing the significance of the query in the decoder, we proposed a heat map initialization query module and a dynamic position encoding module to address the limitations of random initialization. Additionally, we introduced an auxiliary noise query module to stabilize positive and negative sample matching and expedite convergence. Experimental evaluations conducted on the nuScenes dataset validate the efficacy of our approach in enhancing overall detection performance.

## Figures and Tables

**Table 1:** Overview of the nuScenes dataset.

Dateset	Camera	Radar	Boxes	Train	Test	Val	Class
nuscenes	6	5	1.4 M	28,130	6008	6019	23 (10)

**Table 2:** Performance comparison for 3D object detection on nuScenes test dataset. ‘C’ and ‘R’ respectively refer to camera and radar. V2-99 and R101 are VoVNet-99 and ResNet-101. ↑ indicates higher is better, and ↓ indicates lower is better. The same role appears in the table below.

	Split	Modality	Backbone	NDS ↑	mAP ↑	mATE ↓	mASE ↓	mAOE ↓	mAVE ↓	mAAE ↓
RCBEV [35]	test	C+R	R101	0.486	0.406	0.484	0.257	0.587	0.702	0.140
CenterFusion	test	C+R	DLA34 [36]	0.449	0.326	0.631	0.261	0.516	0.614	0.115
MVFusion [37]	test	C+R	V2-99 [38]	0.517	0.453	0.569	0.246	0.379	0.781	0.128
CRAFT	test	C+R	DLA34	0.523	0.411	0.467	0.268	0.456	0.519	0.114
IRBEVF	test	C+R	R101	0.535	0.430	0.622	0.261	0.393	0.377	0.139
IRBEVF-Q	test	C+R	R101	0.575	0.476	0.557	0.241	0.365	0.335	0.132

**Table 3:** The 3D detection results on nuScenes val set.

	Split	Modality	Backbone	NDS ↑	mAP ↑	mATE ↓	mASE ↓	mAOE ↓	mAVE ↓	mAAE ↓
RCBEV	val	C+R	R101	0.497	0.381	0.526	0.272	0.445	0.465	0.185
CenterFusion	val	C+R	DLA34	0.453	0.332	0.649	0.263	0.535	0.540	0.142
MVFusion	val	C+R	R101	0.455	0.380	0.675	0.258	0.372	0.833	0.196
CRAFT	val	C+R	DLA34	0.517	0.411	0.449	0.276	0.454	0.486	0.176
IRBEVF	val	C+R	R101	0.531	0.419	0.653	0.271	0.342	0.330	0.188
IRBEVF-Q	val	C+R	R101	0.568	0.457	0.543	0.266	0.342	0.315	0.139

**Table 4:** Per-class comparisons on nuScenes val set. ‘C.V.’, ‘Ped.’, ‘M.C.’, and ‘T.C.’ denote construction vehicle, pedestrian, motorcycle, and traffic cone, respectively. CenterNet, CRAFT-I, and DETR3D, are camera baselines of CenterFusion, CRAFT, and IRBEVF.

Method	Input	Car	Truck	Bus	Trailer	C.V.	Ped.	M.C.	Bicycle	T.C.	Barrier	mAP
CenterNet	C	48.4	23.1	34.0	13.1	3.5	37.7	24.9	23.4	55	45.6	30.6
CenterFusion	C+R	52.4 (+4.0)	26.5 (+3.4)	36.2 (+2.2)	15.4 (+2.3)	5.5 (+2.0)	38.9 (+1.2)	30.5 (+5.6)	22.9 (−0.5)	56.3 (+1.3)	47 (+1.4)	33.2 (+2.6)
CRAFT-I	C	52.4	25.7	30.0	15.5	5.4	39.3	28.6	29.8	57.5	47.8	33.2
CRAFT	C+R	69.9 (+17.2)	37.6 (+11.9)	47.3 (+17.3)	20.1 (+4.3)	10.7 (+5.3)	46.2 (+6.9)	39.5 (+10.9)	31 (+1.2)	57.1 (−0.4)	51.1 (+3.3)	41.1 (+7.9)
IRBEVF	C	69.1	36.4	45.6	19.3	12.3	48.7	42.7	44.0	58.0	49.8	41.9
IRBEVF-Q	C+R	71.6 (+2.5)	41.7 (+5.3)	50.4 (+4.8)	22.2 (+2.9)	13.3 (+1.0)	53.7 (+5.0)	46.6 (+3.9)	45.5 (+1.5)	60.1 (+2.1)	53.8 (+4.0)	45.8 (+3.9)

**Table 5:** Results of ablation experiments with different modules of the encoder.

	Image	Radar	BEV Fusing Encoding	Projection Fusion	NDS	mAP
(a)	✓	-	-	-	0.425	0.346
(b)	✓	✓	-	-	0.459	0.350
(c)	✓	✓	-	✓	0.475	0.380
(d)	✓	✓	✓	-	0.531	0.430

**Table 6:** Results of ablation experiments on different modules of the decoder.

	HGQI	DPE	ANQ	NDS
(a)	-	-	-	0.531
(b)	✓	-	-	0.538
(c)	-	✓	-	0.540
(d)	-	-	✓	0.544
(e)	✓	✓	-	0.551
(f)	✓	-	✓	0.559
(g)	-	✓	✓	0.553
(h)	✓	✓	✓	0.568

**Table 7:** Performance and parameters of different numbers of BEV features.

BEV Features	10,000	20,000	30,000	40,000	50,000
NDS ↑	0.505	0.411	0.415	0.417	0.416
Params ↓	63 M	65 M	67 M	71 M	74 M
Memory ↓	10.7 G	14.2 G	17.4 G	21.1 G	24.1 G

**Table 8:** Performance and parameters of different decoding layers.

Decoder Layers	1	2	3	4	5	6	7
NDS ↑	0.398	0.485	0.532	0.554	0.565	0.568	0.566
Params ↓	63.8 M	65.3 M	66.7 M	68.2 M	69.7 M	71.2 M	72.8 M
Memory ↓	15.6 G	16.6 G	17.7 G	18.9 G	20.0 G	21.1 G	23.1 G

## Data Availability

The data are contained within the article.
